# Post-release monitoring pathway for the deployment of gene drive-modified mosquitoes for malaria control in Africa

**DOI:** 10.1186/s12936-024-05179-4

**Published:** 2024-11-20

**Authors:** Dorington O. Ogoyi, Julia Njagi, Willy Tonui, Brinda Dass, Hector Quemada, Stephanie James

**Affiliations:** 1 Department of Biochemistry and Biotechnology, Technical University of Kenya, P.O BOX 52428, Nairobi, 00200 Kenya; 2 National Biosafety Authority, P.O. BOX 28251, Nairobi, 00100 Kenya; 3 African Genetic Biocontrol Consortium (AGBC), Nairobi, Kenya; 4 GeneConvene Global Collaborative, Foundation for the National Institutes of Health (FNIH), North Bethesda, MD USA

**Keywords:** Malaria, Gene drive, Mosquito, Protection goals, Regulation, Post-release monitoring, Concerns, Genetic biocontrol

## Abstract

**Background:**

Gene drive-modified mosquitoes (GDMMs) have been promoted as one of the innovative technologies that may control and eliminate malaria and other mosquito-borne diseases. Several products are in early stages of development, targeting either population suppression or population modification of the mosquito vector. However, there is no direct experience of conducting risk assessment for environmental releases and subsequent policies regarding conditions for post-release. This study was carried out to gain additional insights on the possible post-release concerns that may arise, as they may inform future risk assessment and planning for deployment.

**Methods:**

This study involved desktop reviews on post release monitoring experiences with previously released biological control products. Stakeholder consultations involving online surveys, and face to face workshop with experts from selected African countries from Eastern, Western, and Southern African regions was then carried out to establish post-release monitoring concerns for GDMMs.

**Results:**

Review of genetic biocontrol technologies showed only limited lessons from post-release monitoring regimes with a focus largely limited to efficacy. For genetically modified organisms general surveillance and case-specific monitoring is expected in some of the regions. A number of post-release monitoring concerns in relation to the protection goals of human and animal health, biodiversity, and water quality were identified.

**Conclusion:**

Based on established- protection goals, several post-release monitoring concerns have been identified. Subject to a rigorous risk assessment process for future GDMMs products, the concerns may then be prioritized for post-release monitoring.

## Background

The deadliest malaria parasite in humans is *Plasmodium falciparum*, which is most prevalent in the African continent, representing a major public health threat in endemic areas. Transmission is exclusively through the bite of an infected female mosquito. Of the 3500 or so mosquito species that exist, only those within the *Anopheles* genus are actually capable of transmitting human malaria. Among these, about 40 species are at a level of major concern to public health [1, 2]. *Anopheles arabiensis*, *Anopheles coluzzii,* and *Anopheles gambiae* from the Gambiae complex and *Anopheles funestus* from the Funestus complex are the major extant malaria vectors in Africa [1, 2].

According to the World Health Organization (WHO) 2023 World Malaria report [3], there were an estimated 249 million malaria cases globally in 2022, in 85 malaria endemic countries. Ninety-four percent of those cases occurred within the WHO African Region. The report also estimated global mortality from malaria in 2022 to be 608,000 with the African region accounting for 95% of malaria deaths. Current mosquito control strategies, including long lasting insecticide-treated bed nets, chemical insecticides, and environmental management, are losing effectiveness against this disease due largely to increasing genetic and behavioral vector resistance to these interventions. Thus, new, more effective control strategies are urgently needed to address mosquito-borne diseases (MBDs) [3].

Among such innovative vector control strategies is the idea of genetic biocontrol, the deliberate introduction of genetic traits into a mosquito population that will reduce its ability to transmit disease agents, either by suppressing its numbers (population suppression) or by affecting the intrinsic capacity of the insect to host the disease agent (population modification) [4, 5]. Genetic bicontrol strategies involving gene drive have been suggested to have great potential in the control and elimination of malaria and other mosquito borne diseases [5]. Gene drive has been defined as the phenomenon of enhanced inheritance in which the prevalence of a genetic element or alternative form of a gene increases in subsequent generations, even in the presence of some fitness cost [6].

There are two primary strategies for deploying gene-drive systems to reduce the disease impacts of insect-borne pathogens. Population suppression introduces deleterious traits into a population, leading to those populations being eliminated or much diminished. If the mosquito population is sufficiently reduced, disease transmission will be halted. Mathematical modelling of suppression drives with differing fitness costs predicts that an equilibrium level in populations of an infinite size will be determined by the copying efficiency of the drive element [7]. The second approach is to modify the insect vector to prevent it from transmitting the pathogen one wishes to eliminate. This ‘population modification’ leaves the insect in place in the environment but blocks disease transmission. Modification drives are predicted to remain stable in the population for a long period (2–5 years) in the case of *Aedes* mosquito, thus achieving and maintaining local elimination of the pathogen, and allowing public health officials to consolidate their gains [8]. Gene drive technologies can be further characterized according to their anticipated persistence. Self-sustaining gene drives are expected to maintain the genetic modification at high frequency indefinitely within the target population, whereas with self-limiting or localizing drives the extent of the modification is intended to be temporally or spatially constrained [5].

The African Union High Level Panel on Emerging Technologies (APET) identified gene drive for malaria control as a priority area for research for the region among other technologies. African countries were encouraged to increase their participation in gene drive research and ensure readiness for managing such technologies [9]. In anticipation of the development of GDMMs, a guidance framework for their development has been provided [5, 10, 11]. This describes a pathway from contained research through post-implementation monitoring. While no product is envisaged soon, there are several products at the indoor cage stage of experimentation [12–16].

Currently there is no direct experience of conducting risk assessment for environmental releases of GDMMs or other gene drive-modified insects [17–19]. However, there is a very strong opinion that risk assessment for GDMMs can build on existing risk assessment frameworks for genetically modified insects without engineered gene drive; and can also be informed by experience with releasing insects for biological and genetic disease vector/pest control [5, 20]. The development of new or additional guidance for the risk assessment of environmental release of GDMMs has been proposed with specific areas where further guidance may be required to ensure an appropriate level of safety [4, 17, 18, 20]. However, the development of additional risk assessment guidance that is useful and practical is a challenge due to the varied opinions of different stakeholders regarding environmental releases of GDMMs [21–23]. In addition to supporting the need for the development of additional and more practical risk assessment guidance to ensure appropriate levels of safety and to manage perceived risks, Devos et al. [24] proposed other measures including ensuring a more dynamic interplay between risk assessment and risk management to manage and reduce uncertainty through closely interlinked pre-release modelling and post-release monitoring. The authors also identified the need to provide stakeholders with opportunities for active engagement in the risk analysis process. To test the applicability of existing frameworks, the first stage of environmental risk assessment, problem formulation, has been conducted for candidate gene drive-containing mosquito products, to identify potential harms to protection goals for the environment, as well as human and animal health [25, 26].

To engage a wider group of stakeholders, several surveys have been carried out to document the concerns from the target African countries on deployment of GDMMs for malaria control and elimination. While these studies have been carried out using hypothetical population suppression and population modification scenarios, insights have been gained on some of the issues that will have to be addressed before any deployment of such technologies (e.g., [27–29]). None of these studies have investigated mechanisms for post-release monitoring of concerns arising in risk assessment, despite this being cited as an important component of the development pathway [5, 30]. This study begins to address this deficit by eliciting additional input from a range of African stakeholders as well as consulting relevant existing legislation, to identify issues for consideration in post-release monitoring that will further inform planning for eventual deployment of GDMMs. The study involved desktop reviews, online surveys, and face-to-face workshop with stakeholders from selected African countries.

## Methods

In order to understand the possible expectations for post-release monitoring of GDMMs, desktop reviews of legislation and publications on post-release monitoring requirements for genetically modified organisms (GMOs) in targeted African countries and post-release monitoring of other genetic biocontrol technologies was carried out. The biocontrol technologies surveyed included classical biocontrol, sterile insect technique (SIT), *Wolbachia*-infected mosquitoes, and genetically modified mosquitoes. Online surveys and a face-to-face meeting with participants from the targeted countries were also carried out.

For the online survey, participants from the following regions were engaged; Eastern Africa (Kenya, Uganda, Tanzania, and Ethiopia), Western Africa (Ghana, Nigeria, Burkina Faso, and Mali), and Southern Africa (South Africa and Zambia). Invitees were from any of the following three categories: regulators (from biosafety agencies, public health institutions, environment agencies), scientists (expert reviewers, malaria/vector biology researchers, entomologists, or biotechnologists/biologists) and others (religious/community leaders, journalists, or social scientists). Contacts from the biosafety agencies were initially used to identify respondents. Other contacts were made through professional bodies and identified individuals from listed country specific institutional websites. Out of 100 invited respondents, 46 participated in the survey. Participants in the survey included; biotechnologists/biologists (30.4%), regulators (26.1%), entomologists (15.2%), vector/pest control researchers (2.2%); risk assessors (8.7%); journalists (2.2%), and others (13%). The institution of affiliation of these respondents was; researcher/academia (50%); regulatory agency(30.4%); non governmental organization (NGO) (8.7%), international/African wide or regional organization(6.5%), or others (4.4%).

For the online survey, the questionnaire was shared through Google forms and participants were initially given one month to respond. Subsequent reminders were sent with submissions allowed up to the end of the second month. The questionnaire was divided into four parts: (A) personal information, (B) knowledge on malaria, (C) knowledge on GDMMs, and (D) post-release monitoring framework for GDMMs. The section on post-release monitoring framework for GDMMs was based on previously discussed protection goals. Four broad protection goals (biodiversity, water quality, human health, and animal health) were identified as potentially relevant to hypothetical case studies of gene drive to control malaria during previous consultative meetings involving broad expertise from across Africa and elsewhere [27, 28]. Respondents were requested to rank the potential harm in relation to the specific protection goal (1-not at all important; 2-somewhat important; 3-important; 4-very important; and 5-extremely important).

The face-to-face meeting on post-release monitoring concerns about gene drive-modified mosquitoes for malaria control was held during a two-day workshop held in Nairobi, Kenya (18–19th May 2023). Twenty-four participants from the following regions were engaged in the discussions; Eastern Africa (Kenya, Uganda, Tanzania, and Ethiopia), Western Africa (Ghana, Nigeria, Burkina Faso, and Mali), and Southern Africa (South Africa). All the participants had participated in the online survey and were categorized as environmental experts, malaria researchers, biologists, or regulators. The first day of the workshop laid the technical background foundation on gene drive technologies followed by a discussion of the various tools for decision making at the international and national level. The final day was dedicated to group discussion on post-release monitoring issues for GDMMs. The group discussion was preceded by presentations on the post-release monitoring guidelines for GMOs in Kenya and post-release monitoring experience from classical biological control. The rationale and the approach for the current study was then presented to the participants. Participants were then placed in three groups of eight consisting of at least one environmental expert, a malaria researcher, a biologist, and a regulator**.** Individuals from the same country were placed in separate groups. The discussions were based on the four afore-mentioned protection goals [27, 28]. Results from the prior online survey were not disclosed to the discussants.

## Results

### Lessons learned from previous post-release monitoring of other biocontrol products

#### Agricultural biological control

Biological control agents (BCAs) are typically species-specific natural enemies used to control a population of target pest species by debilitating, competing, or killing it, e.g., living predators, parasitoids, competitors, or pathogens. In the agricultural sector, BCAs are used to control weeds and pests that interfere with crop production and lead to losses in crop yields. BCAs are utilized in the agricultural sector because they are safe, self-sustaining, and cost-effective [31, 32].

Three methods can be used to control insect pests using BCA, namely importation (classical biocontrol), augmentation, and conservation of existing natural enemies of pests. Importation involves the introduction of the natural enemies of a pest in a location where they do not occur naturally. A good example of classical biocontrol (CBC) is the control of alfalfa weevils (*Hypera postica*), a forage pest in North America in the 1970s [33]. The pest was accidentally introduced from Europe (its native locale) in the 1910s. The alfalfa weevil was successfully controlled using insect parasitoids with some attacking the larval forms and others attacking the adult stages. Another earlier successful example of classical biocontrol in the 1880s is the control of the cottony cushion scale (*Icerya purchasi*), a citrus tree pest in California, using predatory insect vedalia beetles (*Rodolia cardinalis*) from Australia [34]. Augmentation involves the periodic release of native insect pest enemies in a particular area to boost their population. The releases can be small-scale (inoculative releases) to allow small numbers of the natural enemy to reproduce and establish long-term control of an insect pest. The release can also be large-scale (inundative releases) to allow large numbers of the natural enemy to rapidly suppress the population of a damaging insect pest [35]. An example of inoculative release is the release of *Encarsia formosa* in greenhouses to control the population of whiteflies. Examples of inundative releases are the release of *Trichogramma* species (endoparasitoids wasps of insect eggs) to control moth pests [34, 35], *Bacillus thuringiensis* for control of lepidopteran pests [35] and entomopathogenic nematodes, for the control of insect pests [33]. The third method of biocontrol is the conservation of already existing natural enemies of insect pests. This involves providing environments and habitats to sustain the insect pests’s natural enemies and using low or no insecticide application [36, 37].

Post-release evaluations of BCAs remains a relatively neglected area of study [38]. Post-release monitoring is not mandatory in most jurisdictions and where it is voluntarily carried out, it is usually focused on efficacy. However, post-release monitoring is a pre-requisite for the release of exotic BCAs in some jurisdictions [39] and also recommended in others [40]. According to the North American Plant Protection Organization (NAPPO), post-release monitoring is mandatory in these regions focusing—on species establishment and efficacy of target population suppression. It has been recommended that any release should be followed up by well-replicated sampling and experimental protocols that evaluate the degree of success or failure [41]. These recommendations specify follow-up studies of both efficacy and ecological effects, including (1) landscape scale monitoring across relevant habitat gradients of the abundance of the biocontrol agent, (2) the impact of the biocontrol agent on the target species, (3) the potential for non-target effects, and (4) the response of native species and communities to a reduction in the invasive species. These recommendations have received support from other scientists [42]. According to Porter et al*.* [43], post-release evaluations need not only monitor target population sizes, but also be aware of possible environmental adaptations being displayed as a result of any novel selection pressures that they have encountered.

#### Sterile insect technique

Sterile Insect Technique (SIT) is a biological pest control method based on area-wide releases of sterile male insects to reduce the reproduction in a field population of the same species [44]. SIT disrupts the target organism’s reproductive cycle. Mass-reared males, sterilized using ionizing radiation or chemical treatments, are released in large numbers and may then mate with wild females, resulting in inseminations that either do not produce progeny or produce sterile progeny. SIT has been applied in agriculture, contributing to the management of at least 20 species of insect pests [45]. A veterinary pest, the New World screwworm fly, *Cochliomyia hominivorax*, was eradicated through SIT from both North and Central America and North Africa, where it was accidentally introduced. More recently, SIT success was achieved against the tsetse fly, *Glossina austeni*, vector of animal and human trypanosomiases, which was eradicated from one island of Zanzibar [45]. The first effective applications of SIT in mosquitoes were in the 1960s and 1970s with pilot trials against *Culex quinquefasciatus* [46] and the malaria vectors *Anopheles quadrimaculatus* in Florida, USA [47] and *Anopheles albimanus* in El Salvador, Central America [48].

Application of SIT against some major vector species of *Plasmodium* spp. (malaria) *(An. arabiensis*) and dengue virus (*Aedes albopictus* and *Aedes aegypti*) has been reported. Pilot trials have now been performed on several continents [49]. A recent field trial using a combination of SIT and the insect incompatibility technique (IIT, using the bacterium *Wolbachia*) successfully reduced populations of *Aedes albopictus* in the residential areas of two small islands in Guangzhou, China [50].

The creation of effective and stable sexing mechanisms and the development of effective mosquito mass production and release methods are some of the principal aspects to be developed for the practical integration of these technologies in mosquito control programmes [51]. Aside from the fundamental need to produce large quantities of males, it is essential that the released sterile males have the capacity to survive, actively disperse, and compete for mating in the field. Effective emergence and survival rates together with adequate flight and mating capacities need to be regularly monitored and assured throughout all the sterile insect release programme. Adequate quality control tests supported by standardized procedures need therefore to be developed to effectively measure these parameters and to identify and correct any inappropriate rearing or handling methods affecting the overall male quality [52].

Monitoring the effectiveness of a SIT release is an integral part of any programme [53]. Epidemiological and entomological as well as more general evaluation components for SIT, along with examples of evaluation values have previously been described [54, 55]. The monitoring and evaluation for SIT programmes essentially serves to guide the planning and implementation of the system, measures its effectiveness, and seeks to improve and evaluate the integrated resources [54].

The FAO 2005 standards [56] mentions in particular, the marking of the sterile insects to differentiate them from wild insects, thus enabling monitoring the release of the organisms “in order to evaluate and, as necessary, respond to the impact on the target and non-target organisms”. As a safety precaution, each batch is monitored for radiation dose to ensure reasonable levels of sterilization occur. Otherwise, no additional risk management schemes or post-release monitoring are required, given that the sterilized insects are not able to reproduce and thus any adverse impact is expected to be highly localized and (if required) controllable using conventional (e.g., insecticide) methods [42] or cessation of releases. However, a voluntary study of the environmental impact of SIT for tsetse eradication [57] utilized periodic trapping and assessment of the densities of several non-target species that were abundant in the SIT release area to document a lack of significant effect on local biodiversity.

#### *Wolbachia*-infected mosquitoes

*Aedes aegypti* are well-known transmitters of MBDs such as dengue, Zika, chikungunya, and yellow fever, found in sub-tropical and tropical climatic areas. Dengue is the most significant arboviral disease with an estimated global annual incidence of 96 million cases leading to 40,000 deaths [58]. *Aedes aegypti* are becoming resistant to most common insecticides [59] making it difficult to control in the long term. *Wolbachia*-infected *Ae. aegypti* are being used to inhibit the ability of this mosquito species to transmit MBDs including dengue in Australia, Asia, Latin America, and Southeast Asia [60–64].

*Wolbachia*-infected *Ae. aegypti* are mosquitoes carrying *Wolbachia pipientis*, an endosymbiont bacterium naturally occurring in the cells of many arthropods and some nematodes [62, 65, 66]. The bacteria do not occur naturally in *Ae. aegypti,* but have been stably introduced into them. These bacteria are being used globally in both population modification and suppression programmes to reduce the ability of *Ae. aegypti* to transmit dengue [62, 64, 67]. *Wolbachia*’s effects are based on its ability to induce cytoplasmic incompatibility (CI), a phenomenon that results in *Wolbachia* uninfected *Ae. aegypti* females producing inviable embryos after mating with *Wolbachia*-infected males. The embryos are inviable because *Wolbachia* causes changes in gamete cells [60]. *Wolbachia* mediated population suppression, also known as incompatible insect technique, occurs when large numbers of male *Wolbachia*-infected mosquitoes are released resulting in unproductive mating with wild female mosquitoes [68, 69]. The population suppression strategy involving *Wolbachia* is being used in the United States of America, China, Thailand, and Singapore [50, 68–71].

Alternatively, population modification, also known as *Wolbachia*-mediated pathogen interference, is based on the release of *Wolbachia*-infected male and female mosquitoes. Infected females have a reproductive advantage over uninfected females since they can mate successfully with infected and uninfected males and, because they pass the bacteria to their offspring, can spread the *Wolbachia* through the population. *Wolbachia* infection inhibits replication of a number of arboviruses in the mosquito and therefore reduces pathogen transmission [72]. The first small-scale releases of *Wolbachia*-infected *Ae. aegypti* as a population modification strategy were successfully performed a decade ago in two areas of far northern Queensland Australia [60]. After three years, the researchers did follow-up studies on the release to monitor any changes in the invasion potential of *Wolbachia*. They found that there was a stable pattern of *Wolbachia* invasion [61]. The population modification strategy using *Wolbachia*-infected *Ae. aegypti* technology is now being widely applied in multiple countries to limit transmission of dengue and other arboviruses [62–64, 67, 73, 74].

A number of post-release studies have focused on the stability of the endosymbiont infection in *Ae. aegypti* [75–79]. Breakdown of virus-blocking could be caused by genetic changes in the *Wolbachia*, the mosquito, or the virus. In a recent study, mosquitoes collected from north Queensland had few changes in their *Wolbachia* genome sequences compared to the pre-release strain, indicating a high level of stability to date [80–84]. A comparison of the mosquito genomes also suggested that there have been few changes since *Wolbachia* release [85, 86]. It was also noted that the frequency of *Wolbachia* in mosquito populations has remained high [87, 88] and most host effects of the *Wolbachia* have remained stable [88] with the possible exception of effects on egg quiescence [89].

In a recent study it was estimated that over the next 30 years, there would be a negligible risk of causing more harm due to the release of *Wolbachia*-infected *Ae. Aegypti* [90]. The focus group discussion results indicated considerable feedback, including that ongoing monitoring should be conducted after releasing *Wolbachia*-infected *Ae. aegypti* to prevent hazards identified in the assessment from happening in the natural environment. However, no specific post-release monitoring criteria were identified [90]. There appears to be no formal requirement to collect information during the post-release phase to confirm the assumptions in the risk assessment and its conclusions. However, according to Wimalasiri-Yapa et al. [91], post-release long-term monitoring should include periodic genome sequencing and assessment of the virus, bacteria, and mosquito. Although *Wolbachia*-mediated viral interference has thus far been stable in field-collected mosquitoes [92], dengue viruses have an RNA genome and are subject to relatively high mutation rates compared to DNA-based organisms and microbes, so selection of virus strains which escape from the effects of *Wolbachia* are a possibility [93].

#### Genetically modified mosquitoes

Another technology for controlling MBDs functions similarly to SIT but involves the release of genetically modified (GM) male *Ae. aegypti.* When these GM males mate with wild-type females, their offspring die before reaching adulthood [93]. The first version strains of these GM mosquitoes, created by micro-injecting small amounts of synthetic DNA into mosquito eggs and strain propagation [95], contained a dominant lethal construct that caused the offspring to die at the late larval or early pupal stage unless they were reared in the presence of tetracycline, which turns off the activity of the construct.

The Brazilian National Technical Commission on Biosafety (CTNBio) assessed and approved the release of these GM mosquitoes, and in 2016, the first-ever large-scale release trial of GM mosquitoes was conducted in the city of Piracicaba in the state of São Paulo, Southeast Brazil. About seven million GM mosquitoes were released over a period of two years to achieve suppression levels of 81%[96]. Also in 2016, the U.S. Food and Drug Administration, in connection with an application for field release in Florida, published its preliminary finding that these GM mosquitoes would have no significant negative impact on health and the environment. More recently, a second-generation of this technology has been developed, which limits the lethal effect to female progeny [94, 97]. This second-generation product has been tested and approved for commercial release in Brazil [98, 99]. In May 2020, the U.S. Environmental Protection Agency (EPA) granted the developer, Oxitec, a permit to release these mosquitoes at two U.S. sites and trials are ongoing (https://www.epa.gov/pesticides/following-review-available-data-and-public-comments-epa-expands-and-extends-testing; https://keysweekly.com/42/keys-mosquito-project-enters-a-new-phase/).

Particular requirements have been delineated for living modified organisms (LMO) under the Cartagena Protocol on Biosafety (https://bch.cbd.int/protocol/). The current risk assessment guidelines on GM mosquitoes provide that where a risk has been identified that warrants a response through risk management or where there is uncertainty regarding the overall level of risk of the GM mosquito, risk assessors may consider recommending strategies such as monitoring the GM mosquitoes to ensure that the technology is functioning as intended and to identify unintended adverse effects [100]. However, monitoring policies are not described in this guidance. Therefore, policies set in certain regions or countries can be used as examples that could be useful.

In the EU, post-market environmental monitoring is mandatory in order to trace and identify any direct or indirect, immediate or delayed, or unanticipated effects of a GM organism and or its products [101–103]. The monitoring strategy is further classified as either case specific or general surveillance. Case specific monitoring is defined as being hypothesis-driven and targeted at the assessment endpoints and protection goals identified in the environmental risk assessment as being at risk, or where levels of critical uncertainty were identified in relation to potential risks associated with the GMO [102, 103]. General surveillance is used in some approaches to monitor for effects not anticipated in the risk assessment. Should any such effects be observed, they are studied in more detail to determine whether the effect is adverse and whether it is associated with the deployment of the GMO [102, 103]. Analysis of the Biosafety regulatory frameworks among the African countries targeted in this study provide for post-release monitoring of GMOs with very limited detail on the approach.

Post-release approval conditions have been imposed on GM mosquitoes by CTNBio, the regulatory agency in Brazil. The post-approval plan for Oxitec included monitoring from three representative release sites using traps to evaluate the *Ae. aegypti* and the proportion of the population carrying the transgenic construct. The monitoring was initially to be carried out monthly for twelve months and thereafter, annually to assess the stability of the genetic marker [104]. In addition, the use of tetracycline in Brazil was also to be monitored, through analysis of literature and research reports from wastewater treatment plants, allowing any changes in the use or levels of environmental tetracycline to be detected. Monitoring of the populations of *Aedes albopictus* mosquito was also included in the plan.

### Results of an online survey on post-release monitoring regarding GDMMs

Previous problem formulation exercises [25, 27, 28] identified four protection goals as pertinent to planning for an environmental risk assessment of hypothetical gene drive-containing mosquitoes: human health, animal health, biodiversity, and water quality. Within those categories, certain concerns have commonly been cited as important for consideration in future risk assessments. Here, survey participants were asked to rate these common concerns according to their potential importance for post-release monitoring of a generic gene drive mosquito product.

**Fig. 1:**
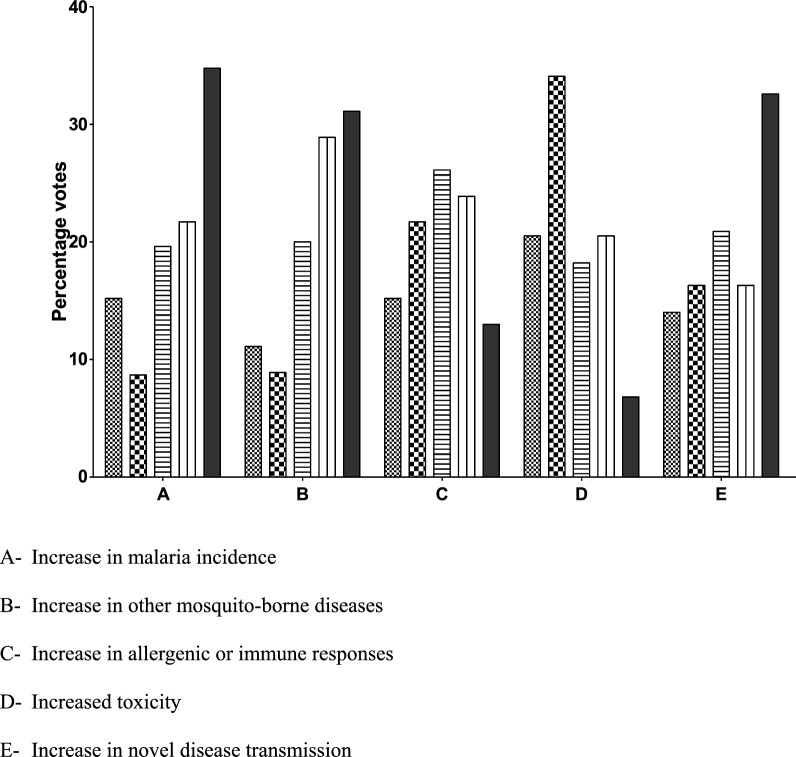
Rating of potential post-release monitoring concern on gene drive-modified mosquitoes with impact on human health. (1—not at all important ; 2—somewhat important  3—Important ; 4—very important; 5—Extremely important ).

**Fig. 2:**
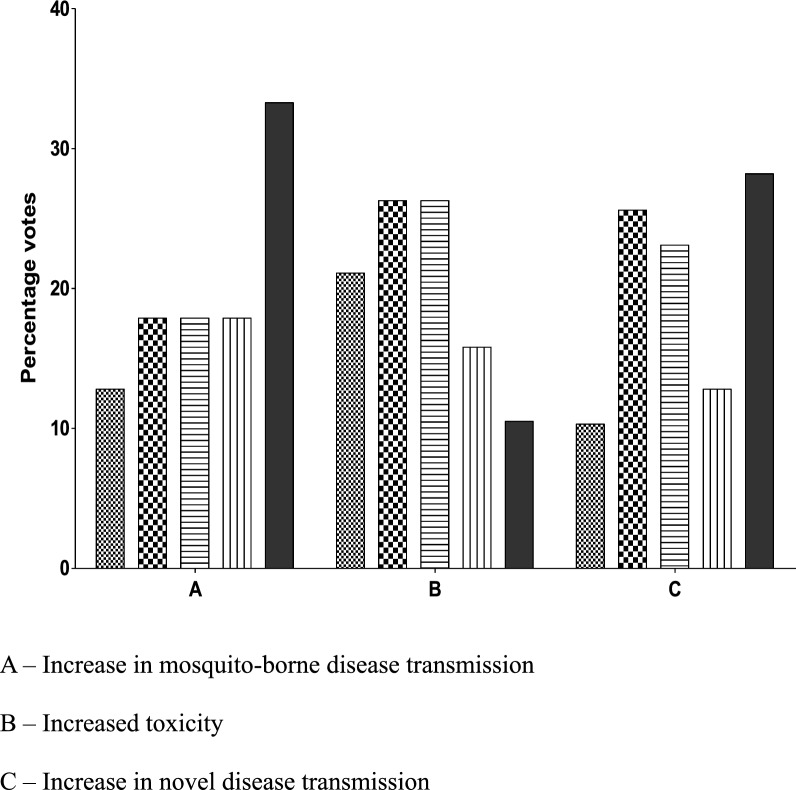
Rating of potential post-release monitoring concern on gene drive-modified mosquitoes with impact on livestock health. (1—not at all important ; 2—somewhat important  3—Important ; 4—very important ; 5—Extremely important ).

**Fig. 3:**
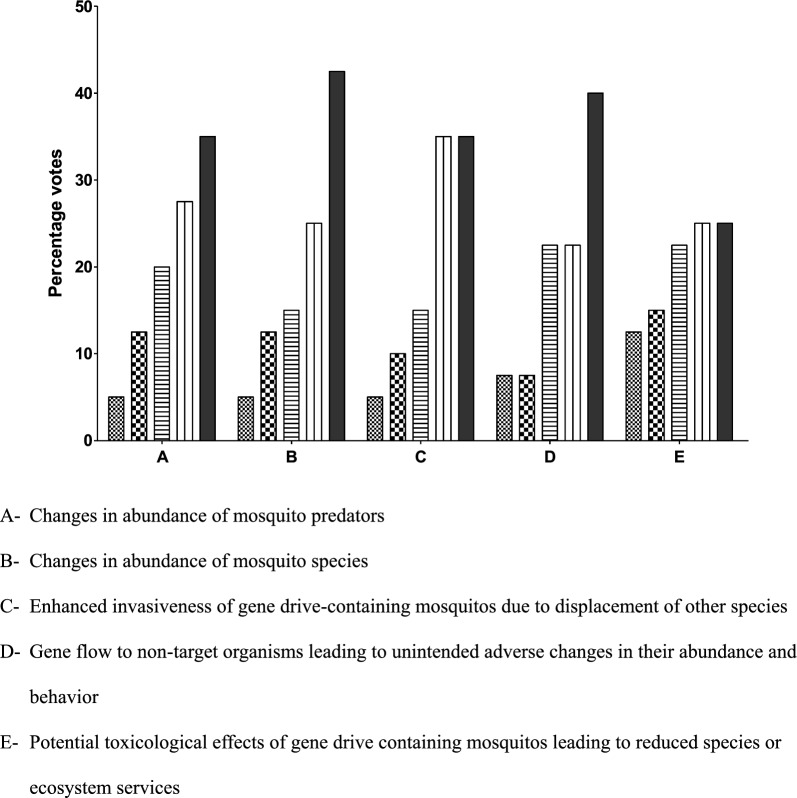
Rating of potential post-release monitoring concern on gene drive-modified mosquitoes with impact on biodiversity. (1—not at all important ; 2—somewhat important  3—Important ; 4—very important; 5—Extremely important ).

**Fig. 4:**
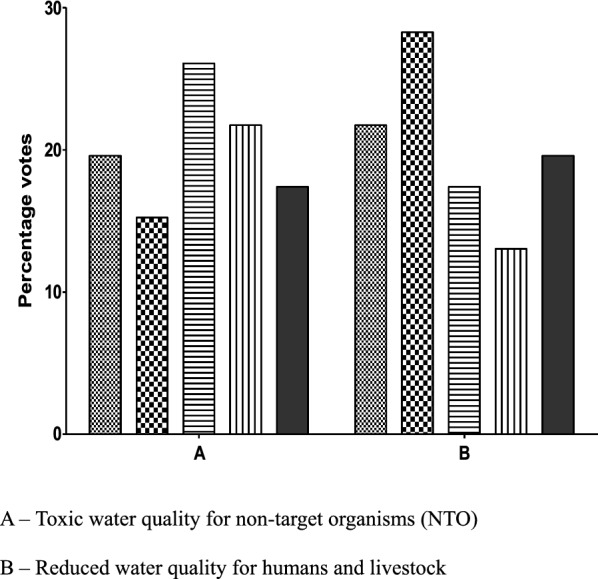
Rating of potential post-release monitoring concern on gene drive-modified mosquitoes with impact on water quality. (1—not at all important ; 2—somewhat important  3—Important ; 4—very important; 5—Extremely important ).

With regards to impact on human health, monitoring parameters identified by the majority of respondents as being extremely important or very important for further consideration were:

(1) increase in malaria incidence, (2) increase in other MBDs, and (3) increase in novel disease transmission (Fig. 1). For the protection goal of livestock health, parameters identified as potentially being extremely important or very important by the majority of respondents were:

(1) increase in MBDs and (2) increase in novel disease transmission (Fig. 2). Regarding possible impact on biodiversity, a majority of respondents ranked the following monitoring parameters as being either extremely important or very important for case-by-case consideration: (1) changes in abundance of mosquito predators, (2) changes in abundance of other mosquitoes species, (3) enhanced invasiveness of gene drive-containing mosquitoes, (4) gene flow to non-target organisms leading to unintended adverse changes in their abundance and behaviour, and (5) toxicological effects of gene drive-containing mosquitoes leading to reduced species or ecosystem services (Fig. 3). Regarding possible impact on water quality, varied opinions were expressed with most respondents rating reduced water quality for humans and livestock as not important or somewhat important while toxic water quality to non-target organisms was rated by the majority as important to extremely important (Fig. 4).

The issue of how long post-release monitoring should be carried out was one of the online survey questions. All the respondents indicated that post-release monitoring would be an important factor for the deployment of GDMMs for the control of malaria. The preferred monitoring period was 5 years (50%). Ten and twenty years for monitoring were rated at the same level (15.8%). Some of the participants indicated that they would prefer post-release monitoring for the period the product remains active (13.2%).

### Results of a workshop on post-release monitoring concerns regarding GDMMs

Participants in the face-to-face meeting were challenged to collectively identify possible post-release monitoring concerns that may arise as a result of the release of hypothetical GDMMs. These discussions also were focused on the four protection goals previously identified as pertinent to risk assessment, but unlike the survey, there was no list of possible concerns to choose from. Additional consideration was given to the nature of evidence that would be required to inform the risk assessment and/or the post-release monitoring plan.

The face-to-face meeting generated twelve potential considerations for post-release monitoring of human health impact (Table 1A). These fall within four main themes; increased malaria transmission or severity in humans (# 2, 4, 5, 11, 12), novel disease transmission in humans (#1), increased ill effects from mosquito biting (#3, 7), and decreased ability to control mosquitoes (#8, 9, 10). Less obvious was the consideration that changes in vector behaviour might result in human behaviour change (#6). For example, if the modified mosquitoes change their feeding times, this might restrict human outdoor activities. The participants also raised concerns about the possibility of the modified vector changing its host preference or responding differently to climate change, leading to changes in malaria transmission dynamics (#10, 11). Unexpectedly, participants also questioned whether elimination of parasites could lead to compromised immune systems (#12, which might impact disease transmission or other aspects of animal welfare.

**Table 1:** **A** Listing of post-release monitoring concerns on gene drive-modified mosquitoes with impact on human health. **B** Listing of post-release monitoring concerns on gene drive-modified mosquitoes with impact on animal health. **C** Listing of post-release monitoring concerns on gene drive-modified mosquitoes with impact on biodiversity. **D** Listing of post-release monitoring concerns on gene drive-modified mosquitoes with impact on water quality

A) Protection goal-human health	
#	Post-release monitoring considerations
1	**Increase in novel disease transmission**: Gene drive-modified mosquitoes may vector other parasites/viruses that initially they could not transmit leading to emergence of new or more virulent pathogen strains
2	**Loss of efficacy**: Resistance of *Plasmodium* parasite to gene drive-modified mosquitoes (particularly relevant to population replacement approaches)
3	**Adverse reactions**: The expressed protein as a result of modification can lead to increased toxicity and allergenicity after mosquito bites
4	**Increase in malaria transmission:** Decrease in one vector could result in other vectors increasing and becoming better vectors, and therefore increasing the intensity of malaria transmission (particularly relevant to population suppression approaches)
5	**Increase in malaria severity:** The parasite mutates to survive in the modified mosquito, to become more virulent or to survive in other mosquitoes or new vectors (Particularly relevant to population modification approaches)
6	**Changes in human behavior**: Changes in vector behaviors as a result of modification may result in undesirable changes in human behaviors
7	**Increase in biting nuisance**: The modification may result in undesirable changes in the mosquito's human biting behaviors
8	**Decreased susceptibility to control:** The gene drive-modified mosquitoes can develop resistance to insecticides /repellants
9	**Behavior change:** Gene drive-modified mosquitoes could be more aggressive and also attack new hosts
10	**Changes in host preference:** Modification may result in undesirable changes in mosquitos’ host preferences, such as an increase in preference for humans versus alternative animal hosts, such as cattle
11	**Changes in malaria transmission dynamics:** The modified mosquitoes could respond differently to climatic changes, resulting in changes in malaria transmission dynamics
12	**Decreased immunity**: Elimination of parasites leading to compromised host immune systems

B) Protection goal-animal health	
#	Post-release monitoring considerations
1	**Increase in spread of arboviral diseases**: Modified mosquitoes change their host preferences and preferentially bite animals more than humans, or they become more competent vectors for arboviruses that are a danger to livestock
2	**New/emerging diseases:** Modified mosquitoes could potentially be vectors of new/emerging diseases
3	**Potential malaria transmission in livestock:** Plasmodium parasite could mutate to survive in modified mosquitoes and potentially become more infectious to livestock. (Particularly relevant to population replacement approaches)
4	**Adverse reactions**: The expressed protein as a result of modification, can lead to increased toxicity and allergenicity after mosquito bites
5	**Increased nuisance biting:** Changes in biting behavior could damage the animals leading to ill health or decreased productivity

C) Protection goal-biodiversity	
#	Post-release monitoring considerations
1	**Ecosystem imbalance**: Leading to; shift on the feeding patterns of known predators (change in the food web**), c**hange in feeding patterns in the modified mosquito larvae, or effect on pollination
2	**Hybridization**: Modified mosquitoes could unintentionally breed with other *Anopheles* species to create previously unknown hybrid species
3	**Invasiveness**: Modified mosquitoes could outcompete other mosquito species in the ecosystem and directly lead to decline in other mosquito species
4	**Genetic diversity:** Genetic restriction reducing genetic diversity among the remaining population of target species
5	**Malaria transmission in non-human primates**: As a result of modification
6	**Increased transmission of arboviral diseases in wildlife**
7	**Population displacement**: Gene drives-modified mosquitoes become able to displace other wild type mosquito species to other areas
8	**Emergence of other *Plasmodium* vectors:** As a result of reduction of *Anopheles* population
9	**Environmental pollution**: Arising from death large number of larvae
10	**Unapproved transboundary movement**

D) Protection goal-water quality	
#	Post-release monitoring considerations
1	**Changes in abiotic water quality parameters:** Changes in mineral content of water due to changes in larval feeding behavior
2	**Increased toxicity**: Toxicity from GDMM larvae dying en masse in the water
3	**Changes in biotic parameters**: Ecosystem imbalance resulting from GDMM larvae in water
4	**Loss of a water quality bioindicator**
5	**Effect on water utility** impact on water quality for recreation

The workshop participants also raised five considerations for monitoring the impact on animal health (Table 1B). Most of these fit under the broad topics of increased disease transmission in livestock (#1, 2, 3,), and altered mosquito biting negatively affecting animal welfare (#1, 4, 5). Under the first topic, major concerns related to the potential for the modification to result in altered vector competence or behaviour. Under the second topic, participants envisioned ways that mosquito biting could be directly or indirectly detrimental to livestock.

The post-release monitoring concerns with impact on biodiversity are listed in Table 1C. In previous classifications of the possible harm to biodiversity, potential impact has focused largely on reduction in density of valued species or ecosystem services [25–27]. Concerns in this area have included such impacts as decrease in mosquito predators, changes in abundance of other mosquito species, and invasiveness in gene drive-containing mosquitoes due to displacement of other species. Effects directly resulting from the transgenic construct may include gene flow to non-target organisms leading to population suppression or potential toxicological/allergenic effects of gene drive-containing mosquitoes leading to reduced species or ecosystem services. While most of the concerns voiced by participants at our workshop related to potential effects on other species or ecosystem services (#1, 2,3, 5, 6,7), the possible fitness cost was raised under concerns #8 and #4. Concern #4 focused on possible genetic restriction, resulting in reduction in the genetic pool of the mosquitoes, while concern #8 raises the possibility of emergence of other *Plasmodium* vectors as a result of the reduction of particular *Anopheles* species. During the discussions two other issues were raised related to environmental pollution (#9) and unapproved transboundary movement (#10). Environmental pollution was raised in relation to the possibility of large numbers of mosquito larvae dying in the environment. This concern was also raised during the discussion on water quality. The concern about transboundary movement was raised due to the likelihood of GDMMs crossing political/jurisdictional borders once introduced into the environment. While this could be beneficial to the neighbouring countries, the Cartagena Protocol on Biosafety (CPB) under Article 17 provides for handling of unintentional transboundary movement and emergency measures. Once a party becomes aware of such occurrence that is likely to have significant adverse effects on the conservation and sustainable use of biological diversity, taking also into account risks to human health, the affected or potentially affected states needs to be notified so as to determine an appropriate response, and initiate necessary action including emergency measures. In case the transboundary movement is considered illegal, then Article 25 of CPB would apply in which the affected country may request the party of origin to take remedial actions at their own cost [30].

Post-release monitoring concerns regarding impact on water quality are presented in Table 1D. The issues raised are either linked to reduced water quality for humans and livestock or toxic water quality to non-target organisms. Some participants noted that the survival of aquatic insects, including mosquito larvae, in water is sometimes used as an indicator of water quality [10] and questioned whether this bioindicator might no longer be applicable after GDMMs release. A few participants also wondered whether the presence of GDMM larvae might cause people to avoid using water sources for recreational purposes.

## Discussion

Broad stakeholder engagement is widely recommended as a crucial means for those engaged in research on gene drive-modified organisms to ensure that any future products will be both beneficial and acceptable to the public [5, 105, 106]. In this regard, multiple efforts have been undertaken to understand stakeholder concerns about the potential risks of GDMMs [20, 27, 28, 107–111]. International workshops have been held to discuss safety and efficacy expectations for GDMMs to enter field testing (e.g., [10, 11]). The current study extends prior work by collecting insights on potential post-release monitoring concerns and requirements for GDMMs particularly in the African context.

The WHO has provided recommendations for post-release monitoring of both efficacy and safety in published guidance [5]. Monitoring for efficacy will encompass considerations of whether the transgenic construct continues to perform as expected and any health claim of the GDMMs is being met [5]. GDMMs have been classified as living modified organisms under the Cartagena Protocol on biosafety (CPB) of the Convention on Biological Diversity (CBD) [112]. Monitoring for biosafety of GMOs largely focuses on the potential for adverse effects to biodiversity or human health [113]. Article 7 of the CBD states that Parties to the Convention shall, as far as possible and as appropriate, monitor the components of biological diversity important for its conservation and sustainable use, and identify activities that are likely to have significant adverse impacts, and monitor their effects through sampling and other techniques. Because of the diversity of gene drive systems under consideration and breadth of conditions under which they may be applied, it is necessary to perform a risk assessment that considers the characteristics of the specific gene drive system and its application on a case-by-case basis [5, 114, 115]. Accordingly, case-specific safety monitoring can be used to generate data to evaluate whether the conclusions of the risk assessment are accurate and whether any risk management strategies in place remain effective. It has generally been anticipated, based on past policies and experience with oversight of public health interventions, that new GDMM product approvals will require the applicant to propose a mechanism for monitoring and reporting of adverse events as well as continued product efficacy following releases [5, 30].

To put monitoring expectations for GDMMs in context, we initially examined the requirements for classical and genetic biocontrol technologies. Post-release monitoring applied to other biocontrol methods currently in use to control agricultural pests or disease causing insects revealed few standard requirements. In general, where monitoring is performed, the emphasis is on observation for ongoing efficacy. For example, monitoring the effectiveness of a SIT release is considered an integral part of any programme [53]. Safety as well as efficacy are considered in monitoring each batch of SIT organisms to ensure reasonable levels of sterilization occur. Otherwise, however, no additional risk management schemes are expected to be deployed after release, given that the sterilized insects are not able to reproduce and thus any adverse impact is expected to be negligible. While the FAO (2005)[56] standard also mentions monitoring the release of the organisms “in order to evaluate and, as necessary, respond to the impact on the target and non-target organisms”, this appears to have been applied only on an irregular and voluntary basis.

Perhaps more relevant to the case of GDMMs are technologies involving release of living BCAs capable of reproducing, establishing, and spreading in the environment. With regards to agricultural biocontrol agents, post-release monitoring is not mandatory in most jurisdictions, and where it is carried out it is focused on efficacy measurement. Although it has been argued that monitoring should also focus on the effect on target and non-target organisms and changes in target/non-target population and community level processes/structures [42], this is not a standard procedure. In NAPPO regions, especially for CBC, post-release monitoring is mandatory but focuses on establishment of the control agent and its efficacy in suppressing target species. In the case of *Wolbachia*-infected mosquitoes, monitoring for ongoing effectiveness is recommended. However, there is no widely applied requirement to collect safety information during the post-release phase [42]. This is presumably based on risk assessments such as those conducted by expert teams in Australia, Vietnam, and Indonesia, each of which concluded that over the next 30 years, there would be negligible risk of causing more harm due to the release of *Wolbachia*-infected *Ae. aegypti* [90, 116, 117]. The Indonesian study did recommend that periodic monitoring be conducted following the release of *Wolbachia*-infected *Ae. aegypti* to prevent potential hazards identified in the risk assessment from happening in the natural environment [90]. Suggestions for periodic post-release monitoring have included possible breakdown of virus blocking that may be linked to virus evolutionary escape, and changes to the mosquito or *Wolbachia* genomes that would affect efficacy [91, 93]. While not formally required, some post-release studies looking at the stability of the *Wolbachia* genome and viral interference voluntarily have been conducted.

Requirements imposed on GMOs by countries that are Parties to the CPB are also highly relevant for GDMMs. According to currently available guidance on risk assessment of GMMs, post-release monitoring may be initiated where a risk has been identified that warrants a response through risk management or where there is uncertainty regarding the overall level of risk of the GMM [100]. Brazil is a Party to CPB and is the first country where GM (non- driving) mosquitoes have been released. Regulatory approval in Brazil of a product for population suppression of *Ae. aegypti* included a post-release monitoring plan exemplary of the case-specific approach [104]. This called for efficacy monitoring to be carried out from three representative release sites using traps to evaluate *Ae. aegypti* numbers and the proportion of the population carrying the transgenic construct. The monitoring was initially to be carried out monthly for twelve months and annually thereafter, to assess the stability of the genetic marker [104]. Additionally, it included analyzing the amount of environmental tetracycline in the release areas, since the drug regulates the activity of the transgene and could impact efficacy, and monitoring for changes in populations of the alternative dengue vector *Ae. albopictus* for possible competitive release.

From the online survey and face-to-face meeting, a number of post-release monitoring concerns were raised by a range of experts across Africa, regarding hypothetical and generic GDMMs. However, it must be emphasized that, as mentioned above, post-implementation safety monitoring would be particularly targeted to any risks identified as non-negligible in a case-specific risk assessment, and therefore the opinions related here will be broader and more general than would be expected for any specific GDMM use case. It also is noted that the respondents represented a subset of African stakeholders expected to have some existing knowledge of technical and regulatory issues relevant to gene drive research, who thus were well placed to anticipate potential concerns at this early stage of GDMMs development. Discussions with other sets of stakeholders, for example engagement with communities likely to be impacted by GDMM release, is an expected component of case-specific risk analysis [5, 118] and may uncover the same and other concerns.

With regards to impact on human health monitoring considerations rated by a majority of the online survey participants as being extremely or very important were; (1) increase in malaria incidence, (2) increase in other mosquito-borne diseases, and (3) increase in novel disease transmission. Concerns rated as extremely or very important with respect to livestock health also focused on (1) increase in mosquito-borne diseases and (2) increase in novel disease transmission. Impacts to biodiversity rated as being extremely or very important were, (1**)** changes in abundance of mosquito predators, (2) changes in abundance of other mosquito species, (3) enhanced invasiveness of gene drive-containing mosquitoes and (4) gene flow to non-target organisms leading to unintended adverse changes in their abundance and behaviour, and (5) potential toxicological effects of gene drive-containing mosquitoes leading to reduced species or ecosystem services. Regarding the possible impact on water quality varied opinions were expressed, with most respondents rating reduced water quality for humans and livestock as not important or somewhat important while toxic water quality to non-target organisms was rated as either important or very important. Considerations for post-release monitoring voiced at the face-to-face meeting included observation for increased transmission of malaria or other mosquito-borne diseases in humans or livestock, increased ill effects from mosquito biting of humans or livestock, reduction in valued species or ecosystem services, and reduced water quality for humans and other organisms.

Post-release monitoring considerations related here largely mirrored the potential harms that previously have been raised in problem formulation discussions with regards to the release of GDMMs [25–28, 109]. Problem formulation is the first stage of environmental risk assessment (ERA), conducted to identify potential harms to identified protection goals. This rigorous scientific analysis defines the overall parameters for an ERA and facilitates the systematic identification of potential harms or hazards, as well as their routes of exposure [100, 102, 103, 119]. Where a particular pathway is assessed to have the potential to harm protection goals following further investigations and characterization of the risk in the ERA, risk mitigation options would need to be subsequently evaluated. It is possible that the unmanaged risk could be considered unacceptable by decision-makers, or they could deem a risk to be acceptable when taking into account the potential benefits of the intervention. It may also be possible to put in place risk management strategies that could be considered sufficiently robust by stakeholders to mitigate any risk identified as above a negligible level. The determination of whether or not a concern should be considered for post-release monitoring can only be determined after completion of an ERA for a particular product and use case by the relevant regulatory agencies. Post-release monitoring may be recommended to mitigate uncertainty, to address assumptions made during the risk assessment, to validate conclusions of the assessment on a wider (e.g., commercial) level of application, and to establish a causal link or pathway between GMOs and adverse effects. Monitoring may additionally be used to evaluate whether risk management strategies are being implemented effectively, including whether those strategies are able to detect potential adverse effects before the consequences are realized. Monitoring can also be applied as a tool to detect effects that were not anticipated in the risk assessment and long-term adverse effects. Decisions regarding the need for and adequacy of monitoring plans ultimately will rest with national regulatory authorities.

The results of this study suggest that, regardless of the disparate potential pathways to harm enumerated in various publications, even in a worst-case scenario post-release monitoring efforts would likely focus on a few major harms. Suggestions for methods to monitor for many of these harms following GDMM release in successive testing phases have been published previously [5, 10]. Should ERA call for additional monitoring of efficacy and safety for human and animal health at the stage of broad-scale implementation, there is good reason to expect that this can feasibly be accomplished as GDMMs are integrated into larger public health platforms and malaria eradication protocols. For example, malaria-endemic countries maintain disease control programmes that can assist in monitoring for changes in human disease patterns. Improvements in vector and disease surveillance are an important component of the Global Technical Strategy for Malaria 2016–2030 [4]. Increasing emphasis on a “One Health” approach for predicting disease emergence [119, 120] could provide a basis for monitoring changes in animal health. Methods for monitoring water quality also are increasingly improving [121, 122]. Of the major topical areas identified, there currently is least consensus on mechanisms for monitoring adverse effects on biodiversity, which is often raised as a particular concern for gene drive-containing organisms that become established in the environment [108] and this subject deserves further exploration. Determining how to attribute causality for any observation of increased harm in the context of multifaceted disease control programmes and in the midst of environmental and social changes [121–123] is also a challenge that other new public health technologies will face in the coming years. Advanced consideration of these issues can help to ensure that appropriate monitoring plans will be developed before GDMM releases take place.

It is important to note that the results from this study largely relied on the feedback from selected stakeholders from Eastern, Western, and Southern Africa. Some of the limitations of the study include; varied levels of understanding of GDMM applications among the participants, and the limited number participants and expertise included in the study in both the online and face-to-face workshops. Another limitation was that the discussions were on GDMMs in general rather than on a specific category of GDMMs. This notwithstanding, the study provides insights on concerns that may be considered during the post-release monitoring of GDMMs in Africa.

## Conclusion

This study has revealed that there are no widely applied standards for post-release safety monitoring of other biocontrol or GM organisms that could be applied to GDMMs, reinforcing the conclusion that risk assessment and risk management must be conducted on a case-by-case basis. Through a consultative process with African stakeholders, we have identified several hypothetical post-release concerns based on established protection goals and found that these potentially fall into a few common topic areas, suggesting their feasibility in serving as a basis for developing appropriate monitoring plans. The hypothetical concerns raised here must be subjected to a rigorous case-specific risk assessment process for future GDMM products, and any concerns that eventually are judged to have above negligible risks or those that require risk management strategies should be prioritized for post-release monitoring. Further work will be needed to identify concerns that require post-release monitoring for a particular product and use case, but advanced planning stimulated by prospective studies such as this can help to prepare for future GDMM releases.

## Data Availability

The survey data are provided in the manuscript.
